# Surgery Triggers Outgrowth of Latent Distant Disease in Breast Cancer: An Inconvenient Truth?

**DOI:** 10.3390/cancers2020305

**Published:** 2010-03-30

**Authors:** Michael Retsky, Romano Demicheli, William Hrushesky, Michael Baum, Isaac Gukas

**Affiliations:** 1 Harvard School of Public Health, BLDG I, Rm 1311, 665 Huntington, Ave., Boston, MA 02115, USA; 2 Department of Medical Oncology, Fondazione IRCCS Istituto Nazionale Tumori, Via Venezian 1, 20133 Milano, Italy; E-Mail: Romano.Demicheli@istitutotumori.mi.it; 3 University of South Carolina, School of Medicine, Columbia, SC, USA; E-Mail: WilliamHrushesky@gmail.com; 4 Royal Free and UCL Medical School, Centre for Clinical Science and Technology, Clerkenwell Building, Archway Campus, Highgate Hill, London, N19 5LW, UK; E-Mail: michael@mbaum.freeserve.co.uk; 5 Breast Unit, Department of General Surgery, James Paget University Hospital, Gorleston, Great Yarmouth, UK; E-Mail: igukas@hotmail.com

**Keywords:** breast cancer, dormancy, surgery induced growth, mammography, chemotherapy, primary antiangiogenic therapy

## Abstract

We review our work over the past 14 years that began when we were first confronted with bimodal relapse patterns in two breast cancer databases from different countries. These data were unexplainable with the accepted continuous tumor growth paradigm. To explain these data, we proposed that metastatic breast cancer growth commonly includes periods of temporary dormancy at both the single cell phase and the avascular micrometastasis phase. We also suggested that surgery to remove the primary tumor often terminates dormancy resulting in accelerated relapses. These iatrogenic events are apparently very common in that over half of all metastatic relapses progress in that manner. Assuming this is true, there should be ample and clear evidence in clinical data. We review here the breast cancer paradigm from a variety of historical, clinical, and scientific perspectives and consider how dormancy and surgery-driven escape from dormancy would be observed and what this would mean. Dormancy can be identified in these diverse data but most conspicuous is the sudden synchronized escape from dormancy following primary surgery. On the basis of our findings, we suggest a new paradigm for early stage breast cancer. We also suggest a new treatment that is meant to stabilize and preserve dormancy rather than attempt to kill all cancer cells as is the present strategy.

## 1. Introduction

We are taught by the scientific method that there are experiments and there are theories. Theories are proposed to explain data and experiments are performed to test theories. When there is satisfactory agreement between theory and experiment we begin to accept that the theory is likely valid and may move on to study other systems. But what happens when there is disagreement between theory and experiment?  It does not matter how long the theory has been around or who has endorsed it, if theory and experiment disagree, we are obligated by the scientific method to reexamine the theory.

In this paper we review our work over the past 14 years that challenges the validity of a main underlying theoretical paradigm that in some important ways guides intervention of breast cancer.  This paradigm is based on the dogma that metastatic breast tumor growth is continuous. If that is not true, as a necessary corollary to discontinuous growth, dormant phases may be only quasi-stable and may be interrupted by an intervention or other external change. We will show that while dormancy is most observable in laboratory environments as delayed tumor growth after cancer cell implant, the sudden escape from dormancy is the most conspicuous effect in clinical breast cancer especially when synchronized. (Note: There are other options, but the traditional way relapse data are presented in clinical oncology is to treat many patients similarly and then graph the fraction who have not relapsed with metastatic disease measuring each from the time they had surgical removal of the primary tumor.  This has the effect in data presentation of artificially synchronizing all patients to surgery even though each was treated at different calendar dates.)  Millions of healthy women have been screened for cancer and cancer patients have been treated by interventions based on the continuous growth assumption. We are of the opinion that interventions should best be guided by better knowledge of how tumors grow.

The burden is on us to first show that evidence in support of continuous growth is not strong. Then we have to show that the growth model that we propose provides better agreement with clinical data. As will be seen, growth description of breast cancer plays an important role in unexpected and far ranging areas of clinical breast cancer. In this paper we will cover these aspects of clinical data and explain in some detail why the continuous model fails and why the new model we have proposed is probably closer to a true description based on its ability to provide explanations to a wide variety of effects that were previously thought to be unconnected.

Just to cast early doubt on continuous growth and to show the importance of quantitatively understanding tumor growth in clinical breast cancer we will discuss a subject of high current interest. Mammographic screening for early detection of breast cancer is a widely accepted and long used tool in attempts to reduce the mortality and morbidity from the disease.  But lately there have been some very public disturbing developments. In the UK in March 2009, a group of scientists, physicians and advocates in a published letter to Times of London criticized the National Health Service for not providing honest information on harms from mammography. They cite the report from the Cochrane organization that 2000 women need to be screened for 10 years to save one additional life from breast cancer but in the process, 10 women will be over-diagnosed and be treated for a condition that does not need to be treated. The National Health Service has agreed to review the information on possible harms provided to women when invited to participate in mammography screening for early detection of breast cancer. This was a significant news event in the UK. A very short time later, in the United States, the U.S. Preventive Services Task Force reversed their previous recommendation and now does not suggest routine mammography for women age 40–49. This was a major news event in the US in November 2009 and caused much consternation. How tumors grow plays a key role in explaining these highly controversial events.

Breast cancers typically start as cells in the milk ducts of the breast that somehow or other become transformed into a state capable of malignant growth. They can grow in the duct and, while remaining there, are called ductal carcinoma in situ or DCIS. Some may ultimately invade the breast tissue and are then capable of producing a tumor that can lead to dangerous breast cancer. However, other cases of DCIS may never invade the tissue in the remaining life of the patient. 

A problem stems from the fact that DCIS cannot presently be classified into those that are dangerous and need to be treated or those that are destined never to invade and can be safely watched or even ignored.  This might never be possible by studying the pathological focus itself as the secret might lie with the host stroma and local cytokine production. (See New York Times feature December 29, 2009) Mammography is quite good at finding DCIS. But then what does one do when that happens? Surgical removal is 100% curative but it may not be necessary. This is not a small problem. Approximately 20% of all abnormal findings in mammography are DCIS and while the number is in dispute, 20% to 50% of DCIS never would invade in the subject’s lifetime. So it turns out that mammography saves lives but there are possible serious harms. Treatment involves surgery and perhaps more extensive therapy if the physician or the patient is sufficiently concerned. The DCIS conundrum is rarely disclosed in public since it is thought that if women were told this they might not opt for mammography. Getting women screened with mammography is a major goal of some organizations so this information is withheld as its release will be contrary to achieving their goal. This is highly patronizing to women. It has been described as “Mommy knows best” as if talking to a child [1].

As other evidence, according to a Danish report, 39% of forensic autopsies of women age 40–49 show clinically occult breast cancer, a number much larger than the lifetime risk of breast cancer of 13% in US and 8% in Denmark [2,3]. Based on this information alone, it could legitimately be argued that mammography should not be routinely recommended for women age 40–49. 

These examples, strongly suggesting that breast tumor growth is not always continuous, well serve to demonstrate the importance of quantitatively understanding tumor growth and what trouble can be caused when medical interventions are guided by dogma rather than by solid data. Health science policy has gotten ahead of health science. It behooves us to better understand how tumors grow.

In the following sections we present arguments as to why we consider the continuous growth model inaccurate and why we consider the new model to better describe clinical breast cancer. In addition to the introduction and conclusion, there are three separate sections to this document that may be considered as adjoining essays. Each was the responsibility of one of the authors. The authors have various medical and scientific backgrounds (and writing styles) that may be apparent. Redundancy of some information was impossible to prevent especially since there are a few early key developments that formed the basis of our ideas and led us along various paths that are presented here.

The phrase “an inconvenient truth” in our title is borrowed from both the poignant message on global warming from Al Gore and a recent paper by Buxton *et al.* [4] proposing a biological mechanism that might explain our findings.

## 2. Models, Cancer Models and the Natural History of Breast Cancer

### 2.1. The Natural History of Breast Cancer

The expression “Natural History” has two meanings. Historically it has come to mean the systematic study of all natural objects, hence the famous collection of dinosaur skeletons, trilobite fossils and Darwin’s specimens from the Galapagos Islands in the Natural History Museum, South Kensington, London. Another meaning to this expression used as a medical term, is the behavior of a disease in the absence of treatment or in other words left to nature.

In the modern world we accept the concept that many minor ailments are self limiting and jokingly reassure our friends that their bad cold will get better in a week, but with whiskey, a warm bed and tender loving care it will only take seven days!

With more serious conditions that are life threatening or could lead to chronic dysfunction we treat in order to influence this natural history in a favorable direction and rely on the history books to tell us what would have happened without treatment but with careful observation alone. Unfortunately in the days before active treatment of serious disease, careful and systematic observation were also exceptional. And this applies in particular to carcinoma of the breast.

Another relevant issue here is that we don’t always see in a dispassionate way the objective reality of that which we observe but more likely a distortion, refracted through the prism of our personal prejudices. Observations that reinforce our prejudices are embraced and those that challenge our beliefs are ignored or rationalized away.

In this section we wish to concentrate on the natural history of breast cancer and the evolution of conceptual models to explain its behavior. We propose that all this is fundamental to improving the lot of our suffering patients for the simple reason that our treatments are the therapeutic consequence of our belief in the underlying mechanisms of disease. In other words belief systems and treatment modalities are two sides of the same coin.

### 2.2. The Nature of Models and Models of Nature

A model car we understand but a model of nature, what can that mean? Let us explain. Models are not just mechanical miniatures of the real thing; they can be anything else which helps to capture the very essence of the subject of our scrutiny. They can be metaphysical, mechanistic, biological or mathematical. Many sports car enthusiasts keep perfect replicas scaled down to 1:1000 on their desks in preference to photographs of their wives and children. This is a mechanical model. Their wives view the contraption as the work of the devil, that if you like is a metaphysical model and finally the mechanical engineer can reproduce the energy of the internal combustion unit and the torque of the transmission system as mathematical formulae. That is a mathematical model.

### 2.3. An Organic Example is the Rose Bush

Easier to handle is the mechanistic model in a children’s primary school botany book. Here the rose is built up of petals, sepals, stamens, filament, anther and carpel all connected to a stalk with leaves, thorns and roots. It loses its poetry when broken down this way. Even more so when the reductionists do their worst and the rose is described as a molecular model. Curiously enough, much of the beauty and mystery of the rose reappears in its mathematical model.

The new mathematics of Fibonacci numbers, fractals and Lindenmayer systems allow us to generate beautiful floribunda on our computer screens thus linking the mathematical model of the rose to the greater symmetries and complex patterns in nature [5].

### 2.4. The Natural History of an Automobile and a Rose

Left to nature an automobile will rust and its engine will seize up. As our knowledge of the automobile and of the mechanism of rusting developed in tandem we have a ready explanation for this process, which is well understood.

The chemical reaction between iron and oxygen in the presence of moisture leads to corrosion and the production of iron oxide. We can influence this natural history by keeping the car dry, well oiled and locked away. With luck the automobile will now last us up to 20 years - its maximum expectation of life.

The rose has a different and much more complex natural history. Left to nature it will enjoy an annual cycle of renewal, flowering every summer, resting every winter and springing into bud each spring. In addition, left to nature, it grows into angry knots; it develops suckers with seven leaves instead of five on each stem, which grow to prodigious lengths. Then holes appear in the leaves, brown patches of rust add to their disfigurement and green flies infest and destroy the buds. In the bad old days you could accuse your neighbor of witchcraft for blighting your bushes (a metaphysical model of disease) but in this modern era we know that the “rust” is a fungus (Puccinia basdiomycetes) and the holes are thanks to the caterpillars. We can influence this natural history with the aid of scientific horticulture, by pruning in February, putting phosphates down in March and spraying with inorganic chemicals all summer. 

### 2.5. The History of Ideas Concerning the Nature of Breast Cancer

After that long preamble, the relevance of which will become clear, we wish to return to the natural history of breast cancer. If left untreated what would happen and of equal importance, why? 

In addition to its sensual beauty, the breast was revered by the ancients as closely associated with menstrual bleeding, pregnancy and child nourishing or nothing short of propagation of the species [4]. Aristotle stated that “menses goes to the breasts and becomes milk“.

The very earliest record of breast tumors is from the Egyptian Pyramid Age (3000–2500 BC). It is believed that Imhotep, the earliest known physician wrote: “If you examine a man (we are also seeing an early case of gender bias) having bulging tumors on his breast, and you find that they have spread over his breast; and you place your hand upon his breast tumors and you find them to be cool, there being no fever at all therein when your hand feels them; they have no granulations; contain no fluid; give rise to no liquid discharge, yet they feel protuberant to your touch, you should say concerning him: this is a case of bulging tumors I have to contend with.”

This elementary differential diagnosis rules out inflammatory diseases or abscesses. As for treatment: “There is no treatment.”

Today all physicians take the Hippocratic Oath. While Hippocrates (400 BC) made little mention of breast cancer, he noted that it was better to give no treatment for deep seated tumors because treatment accelerated the death process, but if one omitted treatment, life might be prolonged. This is of course relative to the 18 year average life span in those times. There were three precepts established by Hippocrates: those diseases that are curable by medicine are best, those that are not curable by medicine may be curable by the knife, and those that are not curable by the knife may be curable by fire. Those incurable by fire are incurable. It is doubtful that surgery to cure a breast cancer was ever recommended by Hippocrates.

While not a physician, Aulus Cornelius Celsus (30 BC to 38 AD) wrote extensively about medicine and science. Celsus was opposed to surgery and cautery for advanced breast cancer. If in doubt, he recommended local application of caustics. If there was some improvement, then one could conclude that it was not cancer and then consider surgery or cautery. If there was no improvement, one should conclude that it was cancer and withhold treatment so as not to hasten demise of the patient. 

Moving forward 200 years, we encounter Galen of Pergamum (131 to 203 AD) who first associated cancer with the crab and was probably responsible for the first routinely successful surgical treatment of cancer. “Just as a crab has legs on both sides of his body, so in this disease the veins extending out from the unnatural growth take the shape of crab’s legs.”

He further states: “We have often cured this disease in the early stages, but after it has grown to a noticeable size no one has cured it with surgery. In all surgery we attempt to excise a pathologic tumor in a circle in the region where it borders on the healing tissue.” Galen was describing how to provide a clear margin.

Ligatures that are used today to tie off blood vessels were not used in Galen’s time since they were thought to spread the cancer. Galen contended that cancer was caused by “humors”, a theory that dominated medicine for 1000 years. Cancer was due to an excess of black bile or humor. 

Paulus Aegineta (7th century) Greek surgeon agreed with Galen that humors caused cancer. He wrote: “... if overheated it is attended with ulceration, and owing to the thickness of the tumor, cancer of the breast is an incurable disease, for it can neither be repelled not discussed; not yielding to purging of the whole body, resisting the milder applications and being exasperated by the stronger ones. The only chance for a radical cure consisted in taking a complete excision of the part.”

Rhazes (841-926), an Arabian physician, warned those who incised a breast cancer would only produce an ulceration and that only if the breast could be completely removed and the parts burned should an excision be done. He approved of cooling the breast if ulcerations have occurred.

Versalius (1514-1564) was one of the first to challenge Galen‘s humor theory. He published an anatomy book in 1543 that marks the beginning of modern anatomy. His study of the breast anatomy paved the way for new surgical techniques to improve breast cancer surgery.

An important contributor to the development of the treatment of breast cancer was Ambrose Pare (1510-1590), a French surgeon who stated: "If the cancer was small, non-ulcerated, and situated in a region where it could be easily removed, the tumor should be excised, but one should go well beyond its boundaries." Large and ulcerated lesions were treated with sweet milk, vinegar, and ointments. Pare called attention to the fact that the primary breast cancer and the axillary extension of the breast were related. This was the first time that the spread of cancer to regional sites was described.

Henry LeDran (1684-1770), a French surgeon, finally ended the Galen thesis of humors. He stressed that cancer was a local lesion in the early stages and spread via lymphatics. LeDran observed that cure was much less likely when lymph nodes were involved.

Velpeau (1856), a Frenchman, advocated bleeding, leeches, purgatives and emetics. He retained the Galenic ideas and used various drugs to destroy the humors. He wrote: “To destroy a cancerous tumor by surgical means is usually an easy matter and but little dangerous in itself; but the question arises, whether such a procedure affords a chance of radically curing the patient. This proposition remains undecided.” He further states: “The disease always returns after removal, and operation only accelerates its growth and fatal termination.” (Note that this is an adumbration of Fisher’s hypothesis that the disease is systemic once it presents clinically. vide infra)

It is to be recalled that all of this was done without the availability of microscopic anatomy. Further, all surgery was performed without anesthesia. Nitrous oxide was described in 1842 and ether was first demonstrated in 1846.  Local anesthesia (cocaine) was first described in 1884. Halsted, a surgeon at the Johns Hopkins Medical School in Baltimore, who first described the radical mastectomy, became addicted to cocaine while researching the drug upon himself.

Surgery at the time was performed without knowledge of sepsis and was often complicated by local and systemic infection. Louis Pasteur advocated heat to destroy bacteria, and Joseph Lister, in 1867, advocated carbolic acid spray to prevent infection. Not everyone accepted the theory and practice of preventing infection. Samuel Gross, a great surgeon of that time in Philadelphia, stated: "Little if any faith is placed by an enlightened or experienced surgeon on this side of the Atlantic in the so-called carbolic acid treatment of Professor Lister."

Schleiden (1838), a German, was among the first to appreciate the signifi­cance of the cell as a unit in plant structure, and Virchow, another German, considered to be the father of pathology, advanced the concept that any normal cell can become a cancer cell as a result of irritation.

Sir James Paget (1814-1899), without the availability of anesthesia or asep­sis, stated: "We have to ask ourselves whether it is probable that the operation will add to the length or comfort of life enough to justify incurring the risk of its own consequences." He had an operative mortality rate of 10% in 235 cases of breast cancer. He believed the disease to be hopeless and stated: “In deciding for or against removal of the cancerous breast, in any single case, we may, I think, dismiss all hope that the operation will be a final remedy for the disease. I would not say that such a thing is impossible; but it is so highly improbable that a hope of its occurring in any single case cannot be reasonably entertained.”

This pessimistic attitude was also voiced by Robert Liston (1794-1847): "No one can now be found so rash or so cruel as to attempt the removal of the glands thus affected whether primary or secondary."

Hayes Agnew (1818-1892), of the United States, resorted to surgery solely for its moral effect. He believed that surgery actually shortened the life of the patient. He was most pessimistic and stated: "I do not despair of carcinoma being cured somewhere in the future, but this blessed achievement will, I believe, never be wrought by the knife of the surgeon."

### 2.6. Breast Cancer in the 19thC and Early 20thC

A treatise by Dr. Gross of Philadelphia published in 1880 [7] provides a clear insight into the status of the disease in the era immediately before the developments in anesthesia and antisepsis which allowed surgeons to attempt a radical cure of breast cancer.  He describes a series of 616 cases, 70% of which had skin infiltration on presentation, which had ulcerated through in 25% of the patients. 64% had extensive involvement of axillary nodes and 27% had obvious supra-clavicular nodal involvement. Accepting that the meager benefits of surgery seldom outweighed the risks in those days, he judged it ethical to follow the natural course of 97 cases who received nothing other than “constitutional support”.

He describes how skin infiltration appeared an average 14 months after a tumor is first detected, ulceration appears on average six months after that, fixation to the chest wall after a further two months and invasion of the other breast if the patient lived on average 32 months after the lump first appeared. The average time for the appearance of enlarged axillary nodes was 15 months in those few cases that presented with an “empty” axilla to start with. 25% of all these untreated cases exhibited obvious distant metastases within a year and 25% after three years with only 5% surviving more than 5 years. 

Since then a number of different series of untreated breast cancer have been reported. For example Greenwood in 1926 [8] described a 6 year follow up of 651 cases of untreated breast cancer with only 60 remaining alive at the end of this period. Daland in 1927 [9] reported a series of 100 patients who were considered inoperable, unfit for surgery or who had refused the offer of surgery. The average duration of life was 40 months for the whole group, 43 months for those deemed operable at diagnosis and 29 months for those deemed inoperable.

The study that has attracted the most attention over the years was that of Julian Bloom published in 1968 [10]. His data came from the records of 250 women dying of breast cancer in the Middlesex Hospital Cancer ward between 1905 and 1933. Of this group 95% died of breast cancer but it should be noted that almost all of them presented with locally advanced or overt metastatic disease. The survival rates from the alleged onset of symptoms were 18% at 5 years, 0.8% at 15 years (remarkably, one person lived 16 years) with a mean survival of about two and a half years. The reasons given for withholding treatment are also worthy of note: old age or infirmity 35%, disease too advanced 30%, treatment refused 20% and early death the remainder.

Although of historical interest we cannot really believe that these studies help to provide a baseline against which to judge the curative effect of modern treatment. Firstly, as with all retrospective uncontrolled series there has to be an element of selection. Why was treatment withheld? It is quite obvious that in the majority of these cases, with the exception of those refusing treatment, they all had an exceptionally poor prognosis to begin with. Secondly, they mostly represent women seeking medical attention at a time in the late 19thC or early 20thC, when many women were content to coexist with their lump in blissful ignorance until they died of old age or were knocked down by a Hansom cab! Next the accuracy of the diagnosis might be called into question in the days before modern microscopy and the widespread adoption of the histological criteria of cancer.

It would of course be inconceivable to suggest we study an untreated group today and the closest approximation we can find comes from a report of the Ontario cancer clinics between 1938 and 1956, just preceding the jump in breast cancer incidence in the developed world [11,12]. Close on 10,000 cases were analyzed accounting for 40% of all new cases arising in the province of Ontario during this period. Amongst this group were 145 well -documented cases who received no treatment of any kind. Although, yet again 100 of these cases were untreated because of late stage of presentation or poor general condition, the rest were unable or unwilling to attend for treatment. A careful note was made of the date the patient first became aware of the lump from which point survival rates were computed. The 5 year survival from first recorded symptom was 35% with a median survival of 47 months. The most surprising figure was a near 70% 5 year survival for the small group presenting with localized disease!

This then raises the inevitable question, is carcinoma of the breast inevitably a fatal disease if neglected [12]? This question is almost impossible to answer with confidence although hinted at by anecdotal evidence. 

### 2.7. The Influence of Surgery on the Natural History of Breast Cancer.

From the popularization of the classical radical mastectomy at the very end of the 19thC [13,14] until about 1975, almost all patients with breast cancer, of a technically operable stage were treated with modifications of the radical mastectomy. To those without commitment to a prior hypothesis, this allowed for new insights about the nature of the malignant process. Before considering this matter it’s worth revisiting the conceptual model that allowed the radical operation to reign supreme for 75 years.

In about 1840, Virchow described a revolutionary model of the disease building on the development of microscopy and postmortem examinations of the cadavers of breast cancer victims [15]. He suggested that the disease started as a single focus within the breast, expanding with time and then migrating along lymphatic channels to the lymph glands in the axilla. These glands were said to act as a first line of defense filtering out the cancer cells. Once these filters became saturated the glands themselves acted as a nidus for tertiary spread to a second and then third line of defense like the curtain walls around a medieval citadel. Ultimately when all defenses were exhausted the disease spread along tissue planes to the skeleton and vital organs.

The therapeutic consequences of this belief had to await the development of anesthesia and antisepsis in the 1880s but were seized upon by Halsted in about 1895 with his complete experience being described in 1932 [16]. Armed with these insights it seemed inevitable that patients would be cured by radical operations that cut away all of the breast, the overlying skin, the underlying muscles and as many lymph node groups compatible with survival.

So convincing were these arguments and so charismatic their chief proponent, the Halsted operation was adopted as default therapy all round the world. At this perspective we are entitled to ask to what extent did the radical operation add to the curability of the disease and what can we learn about the nature of the beast by its behavior following such mutilating surgery? We can also add a third question concerning human nature and our unwillingness to see facts “which almost slap us in the face” * [17]. (* “It is now, as it was then, as it may ever be, conceptions from the past blind us to facts which almost slap us in the face” - WS Halsted 1908)

Unfortunately, only 23% of patients treated by Halsted survived 10 years. The natural response to this failure was even more radical surgery. Internal mammary lymph nodes that received about 25% of the lymphatic drainage of the breast were not removed in the ‘complete operation’ but included in the super radical operations that followed or in the fields of radiation after surgery.

Retrospective studies indicated that more radical operations improved survival [18]. However, in randomized trials that followed later, no benefit could be demonstrated [19]. Thus even when the tumor seemed to have been completely ‘removed with its roots’, the patients still developed distant metastases and succumbed: 30% of node negative and 75% of node-positive patients eventually dying of the disease over 10 years when they were treated by radical surgery alone [20] and with no evidence of “cure” if patients were followed up for 25 years [21,22]. In this latter seminal study by Brinkley and Haybittle, a group of over 700 breast cancer patients, treated by radical surgery alone and followed up for 25 years, continued to demonstrate an excess mortality compared to an aged matched population.

### 2.8. The Biological Revolution of the Late 20thC

Prompted by the failures of radical operations to cure patients of breast cancer, Fisher proposed a revolutionary hypothesis that rejected the mechanistic models of the past [23]. He postulated that cancer spreads via the blood stream even before its clinical detection, with the outcome determined by the biology of tumor–host interactions. Based on this concept of ‘biological predeterminism’, he predicted the following:

(A) The extent of local treatment would not affect survival; and (B) systemic treatment of even seemingly localized tumors would be beneficial and might even offer a chance of cure.

Several pioneers in the field set up randomized clinical trials to test these hypotheses culminating in a series of world overviews [24,25]. Although the “Fisherian” doctrine is now taken as ‘proven’, we must accept that the proof is more in principle rather than in cure. The benefits from systemic therapy are modest, with a relative risk reduction in breast cancer mortality of about 25%–30% overall, which translates to about 8%–10% in absolute terms. As regards the extent of local treatment, many randomized trials have tested less versus more surgery with or without adjuvant radiotherapy.

A recent world overview of these trials [25] concluded that more radical local treatment, surgery or adjuvant radiotherapy does not have any influence on the appearance of distant disease and overall survival with one caveat (vide infra). This is in spite of the increase in local recurrence rates with less radical local treatment, *i.e.*, although radical surgery or postoperative radiotherapy had a substantial effect on reducing local recurrence rates, it did not improve overall or distant disease-free survival. 

The one exception to the theory of predeterminism might be the “success” of the trials of mammographic screening [26]. From this it might be concluded that 25% of breast cancer deaths in women aged 50–69 could be avoided if caught “early” at a sub-clinical stage. Forgetting the arguments about the scientific reliability of these studies or the reliability of the estimate of benefit [27], at best this still only accounts for about 12% of incident cases *i.e.*, failing those cases in women under 50 or over 70.  All the above can be taken as powerful corroboration of Fisher’s theory that metastases of any importance have already occurred before the clinical or radiological detection in about 90% of all breast cancers.

### 2.9. Phenomena that Challenge the Existing Models

Even in the world overview there is one finding that was not completely in keeping with Fisher’s doctrine of biological predeterminism. Radiotherapy does actually reduce the breast cancer-specific deaths by about 3% - only to be counterbalanced by the increased mortality from late cardiac complications in those patients with cancer in the left breast because of radiation damage to the heart. More recently, two randomized-controlled trials evaluated the benefit of postoperative radiotherapy after mastectomy for tumors with a poor prognosis. The radiotherapy techniques in these two studies minimized the dose to the heart. Not surprisingly, there was a reduction in local recurrence rates, but there was also an improvement in the overall 10-year survival rates—9% and 10% [28,29]. Whatever the explanations for the magnitude of effect in these trials, it is clear that more extensive local treatment is not completely ineffective in improving survival. This could mean that local recurrence is a source of tertiary spread, although the metastases arising from the primary tumor at the point of diagnosis exert most of the prognostic influence. Alternatively, and more likely, in our opinion, it might suggest that the additional surgery (usually a mastectomy) might trigger the outgrowth of latent distant metastases. (*vide infra*)

### 2.10. Adjuvant Systemic Therapy Has Only a Modest Effect on Survival

The development of adjuvant systemic therapeutic regimens was based on the kinetics of tumor growth and its response to chemotherapy in animal models [30]. However, the early clinical trials predicted a large benefit and were consequently underpowered to detect the modest ‘real’ benefit. Consequently, there was considerable confusion, with the positive results of some of the early trials being contradicted by negative or equivocal results of others. The overview analysis, however, confirmed that adjuvant systemic therapy can in fact be beneficial [25]. It is the magnitude of benefit that is disappointingly modest—an *absolute* benefit of a maximum of 12% in high-risk premenopausal individuals and of 2% in equivalent-risk postmenopausal individuals is much smaller than anticipated from the experimental models.

The next step taken by medical oncologists was very similar in attitude to that taken by surgeons only a few decades ago, if a little doesn’t work then try a lot! This approach was bolstered by the excellent rate of long-term cure achieved in hematological malignancies. In addition, tumor cell lines showed a log–linear dose response when exposed to alkylating agents [31,32].

Needless to say the high dose chemotherapy with bone marrow rescue was a failure and the least said about this sorry episode in the history of breast cancer the better, yet there may be lessons to learn from the failure of this approach.

### 2.11. When Does a Primary Tumor Seed Its Secondaries?

If we believe that once a primary tumor gains access to the vasculature it starts seeding metastases in a linear or exponential manner, it should be expected that because a larger tumor has been in the body for a longer time, and therefore has had access to the vasculature for longer than smaller tumors, a much higher percentage of patients with larger tumors should present with metastases. This is true to some extent with regard to lymphatic metastases, *i.e.*, there is a correlation of number of involved lymph nodes with the size of the primary tumor. However, this relationship is far from linear. Thus there are small or even occult tumors that have several involved lymph nodes, while many large tumors are found not to have metastasized to the axilla. This discrepancy becomes even more apparent when we consider distant metastases. It would be expected that the proportion of patients presenting with distant metastases would be higher for those with larger tumors as opposed to those with smaller tumors. Nevertheless, in real life a patient presenting with a primary tumor along with distant metastases is uncommon, however large the tumor. At clinical presentation overt distant metastases are very rare whatever the extent of local disease, yet within 2 years of surgery there is a strong correlation between local extent at presentation and distant relapse [33].

How can this be explained without challenging the linear model of breast cancer spread? One explanation would be that although the number of metastases that are seeded by the primary tumor would be linearly related to the tumor size and biological aggressiveness, the clinical appearance of metastases is triggered or accelerated only after the primary tumor has been disturbed or removed. This conclusion may logically derive from a consideration of the pessimistic experiences of ancient surgeons we presented in previous sections. It also is the result of very modern day science using computer simulations to analyze an unexpected bimodal hazard rate of relapse for patients treated only with surgical excision of primary breast tumors [34,35,36]. Hazards are calculated by dividing the number of events in a particular time frame by the number of patients at risk of having those events at the start of the period.

Relapse data from 1173 otherwise untreated early stage breast cancer patients with 16–20 year [35] follow-up display a sharp peak at 18 months, a nadir at 50 months and a broad peak at 60 months with a long tail extending to 15–20 years. Patients with larger tumors more frequently relapse in the first peak while those with smaller tumors relapse equally in both peaks. There is structure in the first peak. A relapse mode within 10 months of surgery is associated with premenopausal node-positive patients. Similar patterns to the Milan data can be identified in some but not all disease-free survival and hazard of relapse databases for untreated patients [37]. 

Based on a computer simulation [38], breast cancer growth often includes periods of temporary dormancy. The second peak is the natural history of the disease. These relapses result from steady stochastic transitions from single cells (dormancy half-life of 1 year) progressing to an avascular micrometastasis (dormancy half-life of 2 years) to a growing lesion that eventually becomes detected as a relapse. The first peak is too sharp to be the result of steady stochastic transitions. Some breaking of dormancy had to occur at surgery to explain the first peak. 

Accordingly, two previously unreported relapse modes comprise the first peak. In the first 10 months, there are relapses due to avascular micrometastases (preexisting at primary tumor detection) that are stimulated to vascularize at surgery. This surgery-induced angiogenesis mode is most prominent for premenopausal node-positive patients in which case over 20% of patients relapse in this manner. The remainder of events in the first peak are single cells that are dormant at primary detection and are induced to divide as a result of surgery. These then must undergo a stochastic transition to an eventual growing metastasis. The first peak comprises 50 to 80% of all relapses depending on tumor size but independent of age. 

The top of the second peak (at 60 months) marks when the benefit of surgery is first seen. That is, the time that it takes a newly seeded malignant cell to become a detectable lesion is so long that the benefit of surgery, that stops the seeding process, does not appear as a reduction in relapses until 5 years have passed in a patient population. This process may be thought of as a metastatic pipeline that is so long that it is fully 5 years after the entrance spigot is turned off before the pipeline is depleted. 

Naumov *et al.* [39] has observed dormant but viable single cells in a breast cancer animal model and Klauber-DeMore *et al.* [40] has observed small dormant micrometastases and growing larger micrometastases in human breast cancer. Folkman and colleagues [41] have reported many examples of dormant micrometastases in animal models. Within the dormant micrometastases there is balance between growth and apoptosis. There are known factors that inhibit angiogenesis and other factors that stimulate angiogenesis. To maintain a dormant state, inhibiting factors locally dominate. If stimulating factors are increased or inhibiting factors are reduced the dormant condition can cease.

It is well documented in the Lewis-lung model that removal of the primary tumor will reduce angiogenesis inhibitors and it is known that after surgery a sharp spike in angiogenesis stimulators and growth factors occurs to aid in wound healing. Thus it is not surprising that tumor angiogenesis and proliferation result after surgery to remove a primary tumor. Thus a likely trigger for ‘kick-starting’ the growth of micro-metastases could be the act of surgery itself. 

The first peak occurs at the same time, whether the tumor was at stage I or stage III. It is only the amplitude of the peak that changes with stage, the later the stage the higher is the peak, but the timing of the signal remains the same.

These phenomena suggest a nonlinear dynamic model for breast cancer, which, like a chaotic system, is exquisitely sensitive to events around the time of diagnosis. It might even suggest that surgery could be responsible for accelerating the clinical appearance of metastatic disease. However, a randomized trial of surgery versus no surgery to prove this would no doubt be judged unethical in the absence of systemic therapy. Nevertheless, such a model is fortuitously available in the setting of randomized trials of mammographic screening [26]. 

Thus with this new perspective we come back to discussing the trials of mammographic screening. In these trials, surgery is delayed in the control group by about 18–24 months (lead-time) so that the first few years offer the comparison between no surgery in the control arm versus surgery in the screened arm. Later years offer the comparison between “late” surgery in the control arm versus “early” surgery in the screened arm. In a meta-analysis of screening trials for breast cancer, it was found that in women under the age of 50 years, there is an early excess mortality in the third year. In women 50 and above, there is no year with a significant excess mortality.  Since the time between relapse and death in breast cancer is approximately 2 years, it is reasonable to conclude that timing-wise surgical-stimulated angiogenesis for premenopausal node-positive patients could account for the excess mortality in the 3rd year of trials. 

There is a very interesting result of extrapolating these ideas. We are saying that for premenopausal women in the absence of surgery, the “natural” process to active angiogenesis delays metastatic growth by approximately 2 years. Also we know that the incidence of breast cancer for women age 30–39 is 0.5% and for women age 40–49, it is 1.5%. Thus, due to this putative effect, 2% of women of childbearing age live 2 years longer and could bear 2 additional children. This could result in a small evolutionary pressure in favor of temporary breast cancer dormancy regulated by inhibition of angiogenesis in premenopausal women. Colon cancer is very rare in women under age 50 but perhaps ovarian cancer should be examined in this perspective as well to see if an equivalent biology is present.

Clearly a new model for breast cancer is needed that takes into account the fine dynamic balance between the tumor and the host, including various autocrine and paracrine factors which influence proliferation, apoptosis and angiogenesis.

### 2.12. A New Model to Explain the Natural History of Breast Cancer

Taking all this into account we would like to develop a new model to explain the natural history of the disease which in addition to explaining the success of the Fisherian model of “biological predeterminism” also explains the clinical observations that fail to fit neatly into the contemporary early detection paradigm. 

First of all, cancer should be seen as a process, not a morphological entity [42]. Individual cancers, while likely to originate from single cells, are constantly adapting to the local environment. There is no single substance or metabolic defect that is unique to cancer. Clonality, previously considered a hallmark of cancer, is neither always demonstrated in malignancy nor restricted to it [43]. 

The cancer cell is largely normal, both genetically and functionally.

The malignant properties are the result of a small number of genetic and/or environmental changes that have a profound effect on certain aspects of its behavior. The three main processes of cancer, growth, invasion and metastasis, have their equivalents in normal tissues. Most cancers are diagnosed by virtue of their morphological or histochemical similarity to the tissue of origin. At the genetic level, with the exception of deletions, all necessary information is preserved, and the defective portion of DNA is relatively small. The key processes of malignancy are genetically controlled by the under or over expression of normal genes and their products that normally serve essential cellular functions. In addition, pathological and autopsy studies have suggested that most of the occult tumors in breast (and prostate cancers) may never reach clinical significance [44,45]. 

Demicheli and colleagues [46] have also provided evidence that a continuous growth model of breast cancer fails to explain the clinical data. In an analysis of local recurrence following mastectomy for patients undergoing regular follow-up, the continuous growth model yielded tumor sizes too large to be missed at the preceding negative physical examinations, and required growth rates significantly lower than those consistent with clinical data. As mentioned before, the continuous growth model also fails to explain the biphasic recurrence pattern seen when hazards of recurrence are plotted for every year after diagnosis.

The new model is based on the concept of tumor dormancy/latency both in the preclinical phase within the breast and later with the micrometastases that seed in the early phase of the natural history of the disease, once the primary focus has developed its microvasculature. The latter remain in a state of both cellular and micrometastatic tumour dormancy until some signal, perhaps the act of surgery or other adverse life event, stimulates them into fast growth. In particular, groups of cells without angiogenic potential can grow but remain small (up to 10^5^ or 10^6^ cells). The metastatic focus may grow quickly if (i) a subset of these cell switches to an angiogenic phenotype and/or (ii) the inhibition of angiogenesis is removed. The model suggests that the metastatic development of unperturbed breast cancer is a sequential evolution from cellular dormancy to micrometastatic dormancy and then from a non angiogenic to an angiogenic state, with stochastic transitions from one state to the next. 

This model may explain the early peak of hazard function for local and distant recurrences in resected cancer patients by combining with the natural metastatic development of unperturbed disease (“the Fisher effect”) with the angiogenic signal following surgery (“the Folkman effect”). It also correlates well with the finding of a modest benefit after adjuvant systemic chemotherapy.

We can now add a new mathematical model to the biological model described above [36]. Breast cancer is like a complex organism existing in a state of dynamic equilibrium within the host, the equilibrium being very precarious and close to a chaotic boundary. Furthermore, the mathematics to describe the natural history of these “organisms” invokes nonlinear dynamics or chaos theory. This model is the first attempt to apply the new mathematics of complexity to make predictions about the factors influencing the natural history of breast cancer that might one day provide a therapeutic window.

Central to the understanding of this model is the pioneering work of Folkman on tumor angiogenesis [47]. As we know, solid tumors cannot grow beyond 10^6^ cells or about 1–2 mm in diameter in the absence of a blood supply [48]. The initial prevascular phase of growth is followed by a vascular phase in which tumor-induced angiogenesis is the rate-limiting step for further growth and provides malignant cells direct access to the circulation [49]. 

In addition to the importance of the microvasculature, we can also visualize these microscopic foci as existing in a ‘soup’ of cytokines, endocrine polypeptides and steroids, with cells interacting with each other and with the surrounding stroma, interpreting competing signals directing the cancer cells in the direction of proliferation or apoptosis.   Such complexity cannot be modeled by linear dynamics, or even a full understanding of the complete catalogue of genetic mutations at the cellular level, because the critical events of multiple cell-to-cell interaction require a thorough understanding of epigenetic phenomena.

What we now have is a new model of the disease that owes its genesis it part to the interpretation of the results of natural history data-bases or clinical trials by way of hazard rate plots rather than Kaplan Meyer curves. We can now see a new signal appearing against background noise that challenges the assumption of linear dynamics in favor of non-linear mathematics or chaos theory [36]. This “signal” is the early peak of hazard for relapse that follows surgery within 12 to 24 months, whereas the near constant hazard thereafter might be the “echo” of the natural history of breast cancer left unperturbed by surgical interference. 

If that is true then the act of wounding the patient creates a favorable environment for the sudden transfer of a micrometastasis from a latent to an active phase. 

The most obvious prediction of the model would be that biological events that are a natural or evolutionary advantage following wounding would as a side effect also favour cancer progression. (It should be remembered in this context that most cancers are the consequence of ageing beyond the age of reproductive activity that in evolutionary terms is neutral). In fact work published in 2004 confirms the similarities in the gene expression of fibroblasts to wounding to those active in malignant disease [50,51]. Or as Weinberg put it “The way that tumours acquire the ability to create complex tissues does not involve their *de- novo* invention of the complex programme of stromal activation. Instead they activate a latent, pre-existing wound-healing programme that is encoded in the normal genome, which they then use as the strategy for constructing their own stroma” [52]. With that in mind, we can note with no great surprise a slew of “downstream” epi-phenomena linking molecular events that favour wound healing to the progression and prognosis of breast cancer. For example HER2 over-expression is closely linked with the expression of VEGF [53] both of which are associated with a poor prognosis. The fluid from surgical drains is very potent in stimulating epithelial and endothelial cells and this is directly proportional to the magnitude of the operation and indirectly to the age of the patient [54]. COX-2 expression is associated with an aggressive phenotype of duct carcinoma in situ [55] and the angiogenicity of circulating malignant cells in the peripheral blood of breast cancer patients predicts for early relapse and resistance to chemotherapy [56]. Finally the paradoxical “curative” effect of adjuvant Tamoxifen might be as much to do with its inhibition of the secretion of VEGF as to its anti-oestrogenic effect. [57]. 

The therapeutic consequences of the new models are almost self evident. The intervention that suggests itself would be anti-angiogenic, and the timing of the intervention would be preoperative, so that at the time of surgery the system is primed to protect against sudden flooding with angiogenic signals. Indeed, some of the success attributed to adjuvant Tamoxifen or chemotherapy might be a result of their anti-angiogenic potential rather than cytostatic/cytocidal effects [58].

Assuming we can protect the subject from the first peak of metastatic outgrowth, we will then have to monitor her with extreme vigilance. By the time the metastases are clinically apparent it is perhaps too late, therefore monitoring the patient with tumor markers and reintroducing an anti-angiogenic strategy at the first rise in tumor markers might prove successful.

In the meantime we can continue to add additional layers of complexity to the simulations of our mathematical model, to help develop alternative strategies for biological interventions to maintain the disease in equilibrium until nature takes its cull in old age. Unlike the hamster lymphoma models of the past, the new model feeds on complexity and becomes closer and closer to simulating the grand diversity of nature.

## 3. Effects of Primary Surgical Removal on the Tumor System

As above recalled, the surgical extirpation of primary breast cancer has been regarded with different attitudes trough the centuries until the twentieth century progress in antisepsis, anesthesia, and surgery fuelled more and more aggressive surgical treatments. The failure of such aggressive operations to cure patients led to a reversion of this trend, and the last quarter century has witnessed a progressive reduction of the extent of surgery, the biological basis of which is embedded in the hypothesis proposed by Fisher.

Further, clinical investigations and mathematical modeling suggested that surgical resection might not always be beneficial [35,38] as, while it favorably modifies the natural history for some patients, it may also hasten the metastatic development for others, by triggering tumor growth. These concepts seem difficult for many to accept. It is worthy, therefore, to carefully review the venerable history of investigations and speculations revealing the context in which these concepts were conceived and structured to explain experimental data.

### 3.1. Earlier Studies: Phenomena Recognized and General Traits Outlined

The capacity of tumor surgical resection to enhance cancer growth at metastatic sites was clearly identified almost a century ago, though the proposed mechanisms were incorrect and incomplete. In experiments of double inoculations of rat sarcomas a retarded growth of a subsequently injected tumor in comparison with the previous (“primary”) tumor was observed [58]. A competition for essential host derived nutrients for tumor proliferation (*athrepsia* hypothesis) was hypothesized, and this phenomenon was labeled “*concomitant immunity*”, assuming that the inhibition resulted from host immune reaction due to the previous tumor [60]. A few years later, implanted tumors, which if allowed to develop naturally, rarely resulted in spontaneous metastases, were found to frequently produce metastases if the primary implant was incompletely excised [61]. Moreover, for a highly metastasizing mouse tumor, incomplete primary tumor excision resulted in larger metastases than those of the control mice not undergoing tumor resection [62]. *Athrepsia* was considered a possible reason of this observation.

During the fourth and fifth decades of the past century, the concept of “dormant tumor cell” was introduced to describe malignant cells which although remaining alive in the tissues for relatively long periods show no evidence of multiplication during the time and yet retain all their capacity of multiplying [63]. The concept was supported by studies on the effect of partial hepatectomy, sham hepatectomy and observation on the development of hepatic metastases [64,65,66,67]. Studies with parabiotic pairs of animals were performed as well. In animals subjected to repeated laparotomies with partial hepatectomy, sham hepatectomy, liver manipulation and chemical hepatic injury, the incidence of metastasis progressively increased to virtually 100%. It was suggested that tumor cells remain in the liver in a viable but dormant state until triggered into growth by some factor or factors. By liver injury, cancer cells may be converted from the state of “peaceful co-existence” with the host to one of active growth, should “conditions” be appropriate. Other investigations added new details [68,69,70,71,72,73,74,75] and about 50 years ago a few main concepts were explicitly stated [68,69].

- A primary tumor of sufficient size inhibits the development and growth of its distant spontaneous metastases.
- Metastases become established and grow prior to the primary tumor becoming large.
- Removal of the primary results in the establishment and rapid growth of large numbers of latent metastases, the majority of which would have been dormant or would have succumbed if the primary tumor had not been removed.
- The growth-stimulating effects on metastases postoperatively are due to removal of primary.

The next years marked an increasing effort to determine the details of the relationship between tumors in different sites, the surgery-driven effects and the tumor dormancy, which it became clear were intimately connected.

The previously adopted *concomitant immunity* hypothesis began to show its limits when it failed to adequately explain several findings (e.g. that the tumor growth at the s.c. injection site may be depressed in mice that received artificial metastases in addition to a s.c. implant, proving that metastases can inhibit s.c. tumor growth without themselves being affected [76]). The immunologic hypothesis was finally rejected when “*concomitant immunity*” was demonstrated in immune-suppressed animals [77,78] and it was concluded that resistance of mice bearing immunogenic and non-immunogenic tumors is mediated by different mechanisms. The resistance to a second tumor challenge in mice bearing non-immunogenic tumor is due mainly to non-immunological mechanisms. The term “*concomitant immunity*” was appropriately changed to “*concomitant resistance*”. 

At the end of this phase of cumulative investigations, it was recognized [79] that a number of observations supported the idea that a tumor is an *integrated*, *organ-like* entity rather than a collection of independent atypical cells. In particular, it was pointed out that the only significant difference between organ growth, organ re-growth after partial resection, and tumor growth is the resetting of the plateau size upward in tumors. Concomitant resistance is an asymmetrical non immunologic phenomenon, where the inhibited tumor is always the smaller tumor and the larger tumor, paradoxically, continues to grow. 

This series of phenomenological studies was concluded by investigating in animal models both tumor cell population kinetics and host survival time [80]. It was found that early surgical excision of the primary s.c. tumor provided some long term “cures” and an increase in lifespan over the untreated controls. Later excision was, however, non-curative and resulted in an increase in the proliferation and growth rate of metastases. This surgery induced stimulation of the metastatic nodules was accompanied by a small but consistent decrease in median lifespan. Artificial metastases were inhibited by the presence of a second subcutaneous implant and the median lifespan of the doubly implanted mice exceeded that of mice bearing intravenous implants only. It was concluded that in mice bearing widely metastasized carcinoma, surgery alone may have a detrimental effect on life expectancy. 

### 3.2. More Recent Times: Looking for the Mechanisms

As the era of breast cancer conservation surgery emerged, the mechanisms of local recurrence following primary tumor removal were center-staged and the effect of surgical trauma was actively investigated. 

A few early reports on growth parameters of double tumor implants [81,82,83] proved that following removal of one of the tumors, changes occur within 24 hr in the proliferation kinetics of the residual focus. There was a transient increase in tumor growth rate and a measurable increase in the size of the remaining tumor and there was also evidence for the presence of a serum growth factor that might be responsible for the phenomenon. The findings thoroughly refute the idea that removal of a primary tumor is a local phenomenon with no other biological consequences. 

It was also proven that surgical trauma of normal tissue promotes implantation and/or growth of circulating cancer cells [84,85,86,87,88] and that extent of trauma influences the metastatic success rate of these circulating tumor cells [84]. It was confirmed that the ability of malignant tissue to respond to surgical wounding of normal tissue is not tumor cell type or even species specific and that the effect is temporary, diminishing as the wounds heal [85,86,87]. Neo-angiogenesis elicited by mechanisms of wound healing is apparently crucial to tumor growth [87] and wound fluids are both directly mitogenic to tumor cells [88] and angiogenic to avascular microscopic tumors [87]. Among the many mediators in wound healing, transforming growth factor beta (TGF-beta) and basic fibroblast growth factor (bFGF) proved to prominently increase tumor growth at an extent nearly similar to wound fluid [89].

Basic aspects of the relationship between primary tumor and its metastases were made even clearer by the experimental studies of Folkman [41,90,91], including evaluation of DNA synthesis, apoptosis, corneal micropocket assay for angiogenesis and newer technologies apt to purify biological molecules. While in mice with an intact primary tumor, metastases appear as microscopic perivascular cuffs or thin colonies of tumor cells on pleural surfaces, after removal of the primary tumor, large highly neovascularized growing metastases may be observed. DNA synthesis is similar in both situations; apoptosis is however significantly diminished in the growing metastases. In a particular experimental setting, when the primary tumor is present, metastatic growth is suppressed by circulating angiogenesis inhibitors (Angiostatin and Endostatin). It may be assumed that primary tumors secrete inducers of angiogenesis and also generate inhibitors of angiogenesis and that when inducers and inhibitors are shed into the circulation, levels of the more labile inducers fall off rapidly whereas levels of the more stable inhibitors create a systemic antiangiogenic environment that prevents small nests of metastatic cells from inducing neovascularization. As a result, these incipient tumors remain small and dormant. Upon removal of the primary tumor, inhibitor levels fall and the previously dormant metastases expand vigorously. The central role of this angiogenesis switch in explaining some features of the metastatic process has been extensively confirmed [91,92,93,94,95,96].

The previously hypothesized single cell dormancy condition has also been identified and studied [97,98]. Elegant quantitative investigations by several sophisticated techniques including in vivo videomicroscopy proved that a large proportion of injected tumor cells persist as solitary dormant cells. These cells can be recovered as viable cells long after they have been administered. It has been further demonstrated, by direct inspection, that single tumor cells reside in metastasis free organs of mice harboring growing metastases in other organs, and, furthermore, that they resume the same proliferative and metastatic capability as their ancestors after rescue and reseeding [98]. The mechanism underlying the single cell dormancy condition is an object of active investigation and findings support the concept that the fate of disseminated tumor cells is fundamentally determined by tissue microenvironmental signals. In particular, a number of data suggest that Extracellular Signal-Regulated Kinase (ERK) and p38 pathways may be involved [99]: high ERK/p38 signaling ratio is apparently correlated to proliferation whereas the opposite occurs in cellular dormancy. 

### 3.3. Findings from Clinical Studies

Findings of the early experiments in rats [59] were paralleled in clinical investigations on host resistance factors where, in autotransplantation tests of cancer cells (in incurable patients), it was found that even when 1 million cells in suspension are injected subcutaneously less than 50% of the transplants “take” [100]. Also, it was observed [101], although on a small series of patients, that surgical removal of bulky metastases of non-seminomatous germ-cell testicular cancer was followed by a sudden and dramatic exacerbation of the disease, thus suggesting that cytoreductive surgery in patients with advanced testicular tumor in some cases may adversely alter the course of the malignancy. 

Even more direct evidence of surgery induced changes of metastasis steady state was achieved in a study of the vascular density in patients with hepatic metastases from colorectal carcinoma undergoing biopsies or resection for synchronous metastases or resection for metacronous metastases [102]. It was found that both peritumoral and intratumoral vascular density were elevated in synchronous metastases from patients with the primary tumor removed compared to synchronous metastases from patients with the primary tumor in situ. More importantly, an increase in vascular density after resection of the colorectal malignancy was observed in biopsies taken from the same patient. 

The study of biological fluids in patients undergoing surgical procedures has also been revealing. Vascular endothelial growth factor (VEGF) increases postoperatively in sera of patients undergoing surgery for lung [103] and gastric [104] cancer. For breast cancer, wound drainage fluid was found to include EGF-like growth factors [105], VEGF [106,107], endostatin [107] and other unidentified proliferation inducers [105] at levels significantly higher than the corresponding serum levels. The concentration of these substances correlate with the amount of surgical damage associated with tumor resection [105]. In particular, wound drainage fluid and postsurgical serum samples stimulated in-vitro growth of HER2-overexpressing breast carcinoma cells [105]. 

Finally, strong direct support for tumor dormancy in breast cancer was recently provided in an investigation on the incidence of circulating tumor cells (CTC) in disease free patients several years following mastectomy [108]. Fifty nine percent of women displayed CTCs. As, after primary tumor removal, CTC half-life is a few hours, it should be concluded that several years after primary tumor removal, clinically silent tumors foci may exist and continuously shed CTCs. 

### 3.4. A New Way to Address the Question

In spite of experimental evidence, the idea that surgical cancer resection has both beneficial and adverse effects upon cancer spread and growth that result from the modulation of tumor dormancy by the resection has continued to cause denial and consternation among clinicians. In all probability, it occurs because of a reductionist perception of cancer as resulting from invading *alien enemies* that need be completely destroyed in order to achieve the cure. In this framework it is difficult to understand that the primary tumor may exert influences upon distant metastases resulting in inhibited proliferation and/or enhanced apoptosis, mimicking the organ homeostasis that maintains the ultimate organ mass following the growth process. A new conceptual approach to cell and tissue functioning has been recently proposed, however, within which tumor homeostasis may be better understood [109]. 

In the classical approach to molecular biology, cell functioning results from the activation of regulatory pathways, which represent a linear chain of causal relationships explaining a particular phenotype. Yet, this conceptual framework is challenged by the findings that there is significant cross talk between pathways at almost every level of the signaling cascade, that the same molecule may control the expression of up to hundred genes and, conversely, a single gene may be controlled by several regulatory proteins. The advent of genomics and proteomics technologies has further emphasized the idea that molecular pathways are just parts of a complex genetic regulatory network (GRN).

Genes regulate each other and, in addition, epigenetic regulations are present. Thus, genes cannot change their activity independently because of interactions introducing constraints to the network organization, with the consequence that only given gene profiles (cell phenotypes) are permitted (are stable). In tissues, cell functionality is conserved under random perturbations from the microenvironment and additionally the cell is able to undergo specific adaptive changes. Phenotype stability may be explained by the fact that the GRN displays a number of stable “attracting” configurations, with a neighboring “basin of attraction”, corresponding to all states from which the system comes back to the “attractor”. However, under certain perturbation spurs, the GRN may undergo an avalanche of significant changes in gene expressions and, following a transient phase, it may reach a new “attractor” (stable profile), thus showing an adaptation process. It should be emphasized that attractors are self-stabilizing discrete states determined by the mutual interactions of the network components. The provocative idea that the genome is a self-stabilizing entity has been recently supported by an analysis of the entropy changes during differentiation of hematopoietic progenitors to derived erythroid and neutrophil cell types [110]. 

The concept that stable states may emerge from interacting elements may also be used at the tissue level, *i.e.*, when interacting elements are cells [109]. Interactions, embodied by molecular or mechanical cell-cell communications, would accordingly generate tissue level attractors, ultimately sustaining tissue and organ homeostasis. Examples of this picture come from normal organs, such as the liver, where changes in some significant parameter may result in large organ rearrangements. Experimental evidence has also been provided in studies performed in 3-dimensional cell culture systems consisting of collagen-I gel, where myoepithelial cells derived from normal mammary gland of mice are co-cultured with luminal epithelial cells and “spontaneously” organize into acini-like structures.

Looking at cancer from the GRN dynamics approach, we may conceive that a rewiring of the network architecture by genetic or epigenetic changes may reshape the attractor landscape, hence allowing the cell to acquire new, self-stabilizing gene expression programs still preserving basic cellular functions. In particular, the characteristic traits of tumor phenotype (proliferation, migration, invasion, angiogenesis, *etc*.) would not be *de novo* inventions, but co-opted from early self-organizing attractors, inaccessible in the normal mature tissue and made re-accessible because of altered attractor landscape. In other words, the new approach basically suggests that most of the tumor hallmarks could be considered normal-like yet de-contextualized processes, due to altered network architecture. This concept is supported by experimental findings indicating that normal cells may display cancer-like behavior and, conversely, cancer cells may regain normal cell traits [111,112,113,114,115]. In particular, neoplastic tissue would not entirely escape general behaviors and would at least in part comply with tissue level attractors. Accordingly, a main perturbation like primary tumor surgical removal may induce a rewiring if the tissue network architecture implying dormant metastasis switch to a growing phase [116]. The pattern of clinical recurrence may be then considered an indicator of this phenomenon, which generates a time frame for the clinical development of the disease. 

## 4. A New Point of View for a Few Uncertain Questions

### 4.1. Mammography Paradox for Women Age 40–49

With the reasonable probability that screening would detect more and more cancers in very early states, it was expected that mammography screening would result in a major reduction in breast cancer mortality. Trials testing this hypothesis were begun in the 1960s and still continue. To avoid a bias, “intent to treat” analyses are done based on invitation to screening rather than those who are actually screened. Individual trials to determine the value of mammography for young women were producing confusing results, so all such data were presented and reviewed at a NIH Consensus Conference in 1997 and in follow-up papers [117,119]. Restricting our discussion to the 7 large trials conducted before the widespread use of adjuvant chemotherapy, for women age 50–59 there was an early appearing 20–30% mortality reduction resultant from the early detection of breast cancer. However, among women age 40–49, there was an early mortality disadvantage during the first 6–8 years after screening started. Afterwards, some advantage appeared in trials and overviews. 

Because of these unexpected and unwelcome results, the trials particularly for young women have been carefully scrutinized and rescrutinized and it was easy to criticize the trials and dismiss the unsettling data demonstrating harm from mammography in some young women. But these data are all we have. If we discard them, we are left to make decisions based on our personal biases [120,121].

We observed that screening and control arms, even if similar due to randomization, have different surgery timing distributions within the natural history of breast cancer development, resulting from the early recruitment of breast cancer diagnoses for the screening arm only. We calculated that surgery-induced angiogenesis as determined by the Milan data would cause 1 early death per 10,000 screened young women in the 2nd or 3rd year after starting screening [120].  The effect accelerates mortality by 2 years since that is the undisturbed half-life of the pre-angiogenic dormant state. This effect would be most apparent in the early years of each trial. Both magnitude and timing agree with the individual trials and overviews of the 7 relevant trials conducted over decades of time and in different countries. 

### 4.2. Highest Sensitivity to Adjuvant Chemotherapy for Premenopausal Node-positive Patients

Following early trials, clinical consensus reports from the years shortly after the introduction of adjuvant chemotherapy for breast cancer (1980 and 1985 National Institutes of Health Consensus Development Conferences) recommended using adjuvant chemotherapy for premenopausal node-positive patients. Only in later years, after careful analysis of much larger trials with longer follow-up, was it determined that adjuvant chemotherapy is of some value in subsets of node-negative disease, or in any patient with positive nodes. The additional curative benefit of adjuvant chemotherapy is approximately 12% for premenopausal node positive patients and in the 2%–6% range for all other categories [122]. 

These findings are consistent with our metastasis model that includes sudden release from dormancy in synchrony to surgery. The rapid growth of micrometastases and corresponding high chemosensitivity occurs just at the time when adjuvant chemotherapy was empirically determined to be most effective [123,124]. However, an avascular micrometastasis and a single tumour cell will reach the clinical level in different times.  A detailed study of the recurrence dynamics following adjuvant chemotherapy with CMF, indeed, demonstrated that the CMF recurrence rate reduction is largely restricted to two specific temporally separate recurrence clusters occurring during the first and third year of follow-up, while the second-year recurrences are weakly affected [125]. The dynamics of both post treatment recurrence risk and CMF effectiveness are similar for both pre- and post-menopausal women, albeit of considerably higher magnitude in pre- than in post-menopausal women. Therefore, the model provides numerically consistent reasons why adjuvant CMF given following surgical resection of the primary cancer is more effective for premenopausal patients than it is for postmenopausal patients. 

### 4.3. Heterogeneity of Breast Cancer and Aggressiveness of Breast Cancer in Young Women

Breast cancer is well known as a disease exhibiting substantial heterogeneity and extreme variability in outcome for patients within single prognostic categories. Our model suggests that the disease course following primary tumor surgical removal basically follows a common pathway with well defined steps (dormant states) and that the pace (time to transition between states) of the common pathway is governed by pre-existing tumor and host traits (risk factors) [116]. The concept that for a given patient a unique mix of tumor and host factors and initial conditions may control the ability of tumor cells to progress through successive dormant states eventually resulting in clinical recurrence may be considered a reasonable explanation for some of this heterogeneity. Therefore, the model provides a frame for a more quantitative detailing of the disease course, *i.e.*, for a measure of its "aggressiveness". For example, breast cancer in young women is often labeled “aggressive” by clinicians. This refers to the high proportion of relapses that appear very shortly after diagnosis of primary disease and ensuing surgery. From our perspective, this term well fits the 20% of premenopausal node-positive patients that relapse within one year of surgery as a result of surgery-induced angiogenesis.

### 4.4. Excess Breast Cancer Mortality for African American Women

There is an excess mortality in breast cancer for African-American (AA) women compared to European-American (EA) women that first appeared in 1970s and has been worsening since [126,127,128,129,130,131]. The differential access to medical care and screening and even disparities in disease management (diagnostic procedures, treatment decisions, *etc*.) are sometimes considered the source of the different race-related outcome. Socioeconomic explanations are indubitably very important, but they are not sufficient because there is a crossover in excess mortality at age 57. That is, AA patients diagnosed under age 57 have higher breast cancer mortality than EA but over age 57, AA have less breast cancer mortality than EA. 

It is known that the average age of diagnosis of African-American women is 46 while it is 57 for European-American women. In the above section on the “mammography paradox” we explained the biological reasons why mammography is more beneficial for postmenopausal women than it is for premenopausal women. Therefore, it should be expected that after the introduction of mammography in the 1970s, there will be mortality advantages to EA over AA. In other words, the mammography screening introduction may be considered as a probe revealing different traits in the host-disease balance in AA and EA, which are reflected by the change in mortality dynamics. Therefore, while it is obviously essential to equalize access to healthcare, solving ethnic disparities may still require understanding and effectively addressing other biology-based racial differences [132].

## 5. Concluding Statements

We have shown indirect but compelling evidence that there is dormancy in breast cancer and that surgery to remove the primary tumor does occasionally break dormancy. The ability to provide explanations for such a wide variety of effects in breast cancer with a single hypothesis to us is a strong hallmark of a valid theory. That is, it is no surprise that the computer simulation agreed with Milan data since it was built to do that. But then, what else does it tell us that we did not know beforehand?

**Figure 1:**
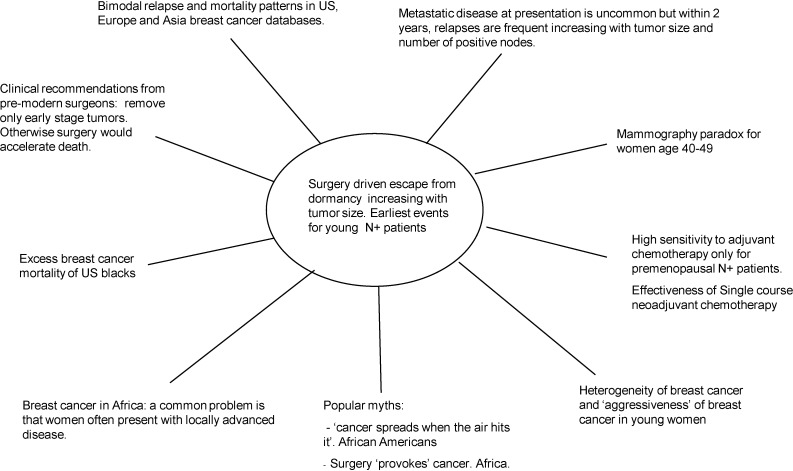
We have suggested that surgery kick-starts growth of dormant micrometastases. This effect increases with primary tumor size and in particular surgery induces angiogenesis in 20% of premenopausal node-positive patients. This one hypothesis seems to explain a variety of previously unconnected effects in breast cancer.

Figure 1 is reminiscent of the parable of the seven wise blind men from Hindustan who are each trying to describe an elephant. One feels the tail and notes that an elephant is a rope. Another feels the leg and says an elephant is a tree trunk, *etc*. From our perspective, the mammography specialist who sees paradoxical results from early detection of breast cancer among young women who then proclaims the trials must have been erroneous, the surgeon who notes that breast cancer in young women is an aggressive disease, and the medical oncologist who knows premenopausal node positive patients respond best to adjuvant chemotherapy are blind to the biology of tumor dormancy and its reawakening by surgical intervention.

Another test if a theory is valid is whether non-obvious predictions based on the theory are correct. The predictions from Demicheli *et al.* Cancer 2007 [126] and the proposed therapy from Retsky *et al.* BMC Cancer 2009 [133] can be considered key testable predictions. Surgery-induced angiogenesis that is mainly restricted to node positive patients who are premenopausal would be the most noticeable in clinical situations since the patient category is small, distinct, and the relapses occur so soon after surgery. Longitudinal or serial imaging coupled with angiogenic biomarker studies started before surgery for young patients who may be node positive would be very useful. Recent detailed analysis of clinical data for individual patients by Hanin and Korosteleva indicating metastases growth acceleration after surgery shows relevant measurements can be extracted from clinical situations [133]. 

We have not proven that the dormant states involved are restricted only to nondividing single cells or avascular micrometastases. Dormancy of these two states and its breaking after surgery are numerically consistent with the Milan data but truth be told, we have not fully examined if other micrometastatic size dormant states might also provide adequate fit to the Milan data. There could be various biological mechanisms preventing tumor growth and terminated by some connection to surgery [57]. Thus the title of this paper is intentionally left vague as to which of the various possible dormant states are perturbed by interventions. As an overall statement, the positive predictive power of our model is impressive and we suggest it should be considered as the leading candidate for the model that best describes how breast cancer progresses.

We have not shown how this model can be combined with cancer stem cell theories. That would be an important development. Cancer stem cells are associated with dormancy so there may a connection as yet unidentified. We have also not shown whether this theory has any relation to Her-2 as a marker for early relapse and benefit from Herceptin. With recent availability of gene chips, there has been much activity with correlating breast cancer relapse or mortality with genetic profiles among patient populations. We would advise that these correlations should be segmented according to the different disease progression patterns that we have identified. That is, just looking for gene assay correlations with survival ignores the multiple biologic pathways that breast cancer can take. We suggest research in this field be along the lines as reported by Zhang *et al.* [135].

Other cancers also need to be carefully examined. There are data showing indications of similar activity especially in melanoma [136] and osteosarcoma [137]. The information provided in this document will help scientists identify dormancy and surgery induced growth in other cancer sites. The ultimate value of this knowledge is to propose new methods of intervention that may reduce mortality and morbidity. We suggest some progress has been made in this regard in breast. 

We have covered much terrain and history in breast cancer. There have been many persons in the 5000 years of breast cancer intervention who have made important contributions but we wish to draw particular attention to Bernard Fisher and the late Judah Folkman. 
